# Sub-RDT *Plasmodium vivax* infections and G6PD deficiency in Kayin State, Myanmar

**DOI:** 10.1186/s12936-026-05952-7

**Published:** 2026-06-01

**Authors:** Aung Myint Thu, Aung Pyae Phyo, Chanapat Pateekham, Saw Hay Blu, May Myo Thwin, Ladda Kajeechiwa, Wanitda Watthanaworawit, Yanada Okkararungrot, Germana Bancone, Gornpan Gornsawun, Paw Khu Moo, Khaung Klain, Pyae Linn Aung, Wang Nguitragool, Wirichada Pan-ngum, François Nosten

**Affiliations:** 1 Shoklo Malaria Research Unit, Mahidol-Oxford Tropical Medicine Research Unit, Faculty of Tropical Medicine, Mahidol University, Mae Ramat, Bangkok, Thailand; 2 Department of Tropical Hygiene, Faculty of Tropical Medicine, Mahidol University, Bangkok, Thailand; 3 Centre for Tropical Medicine and Global Health, Nuffield Department of Medicine, University of Oxford, Oxford, OX3 7BN UK; 4 Mahidol Vivax Research Unit, Faculty of Tropical Medicine, Mahidol University, Bangkok, Thailand; 5 Department of Molecular Tropical Medicine and Genetics, Faculty of Tropical Medicine, Mahidol University, Bangkok, Thailand; 6 Mahidol-Oxford Tropical Medicine Research Unit, Faculty of Tropical Medicine, Mahidol University, Bangkok, 10400 Thailand

**Keywords:** *P. vivax*, G6PD, Sub-RDT infection, Low-density *P. vivax* infections

## Abstract

**Background:**

In Kayin State (Myanmar), *Plasmodium falciparum* incidence declined substantially between 2014 and 2020 following intensive elimination efforts, leaving *P. vivax* as the predominant species. Elimination of *P. vivax* is challenging due to relapses, presence of low density reservoirs and a high prevalence of glucose-6-phosphate dehydrogenase enzyme (G6PD) deficiency in the area, which constrains the use of 8-aminoquinoline drugs for radical cure. This study assessed the prevalence of low-density, sub-RDT (rapid diagnostic test) *P. vivax* infections and G6PD deficiency in 23 villages in Kayin State from 2020 to 2021.

**Methods:**

Participants were screened for malaria using malaria RDT (mRDT), a reverse transcriptase real-time polymerase chain reaction (rRT-PCR), an enzyme-linked immunosorbent assay (ELISA) and their G6PD status was assessed using a quantitative point-of-care test. Prevalence estimates with 95% confidence intervals were calculated and stratified by sex and geographic area and group differences were compared using chi-square or Fisher’s exact tests.

**Results:**

The mRDT detection rate was very low (0.5%), with 26 *P. vivax* and 1 *P. falciparum* infections detected among 5509 individuals. Positivity by rRT-PCR for *P. vivax* was 14.3% (317/2219) followed by *P. falciparum* 8.3% (185/2219) and unidentified *Plasmodium *species infections 0.8% (18/2219). The prevalence of sub-RDT *P. vivax* infections (defined as rRT-PCR positive and mRDT negative) was 14.1% (311/2211). The corresponding figure for *P. falciparum* was 8.3% (184/2218). Median village prevalence of sub-RDT *P. vivax* infections was 15.2% [IQR 4.0–23.6, range 0–40.4] and 6.0% [IQR 2.1–16.0, range 0–27.0] for *P. falciparum*. Hpapun township (north) had a sub-RDT *P. vivax* prevalence six times higher than Myawaddy township (south). The overall proportion of G6PD deficiency among males was 21.7% and 10.6% in females (P < 0.001). G6PD deficiency showed village-level variation, with a median of 14.4% (IQR 12.3–14.8) in Myawaddy and 17.3% (IQR 14.0–23.1) in Hpapun (P = 0.05). No association was observed between G6PD status and sub-RDT *P. vivax* infection in either males or females across townships.

**Conclusions:**

The coexistence of sub-RDT *P. vivax* reservoirs and a high proportion of G6PD deficiency pose a major barrier to elimination. These findings highlight the need for high-sensitivity diagnostics to detect low-density infections and reliable point-of-care G6PD testing to effectively target *P. vivax* malaria in Kayin State, Myanmar.

**Supplementary Information:**

The online version contains supplementary material available at 10.1186/s12936-026-05952-7.

## Background

The Greater Mekong Subregion (GMS) aims to reach *Plasmodium falciparum* elimination by 2030 through regional collaborative efforts. Countries within the GMS, including Myanmar, have achieved significant progress in malaria elimination over the past decade with support from the Regional Artemisinin Initiative (RAI). In Kayin State (Myanmar), the incidence of *P. falciparum* malaria declined significantly after a large and intensive elimination campaign [1] and *P. vivax* became the dominant species. From 2014 to 2020 in Kayin State, annual *P. falciparum* incidence dropped by 90% from 15.9 to 1.6 cases per 1000 persons. Over the same period, *P. vivax* incidence fluctuated but decreased only slightly, from 25.5 to 21.2 [2]. Both species had higher incidence rates in northern township than in the central and southern townships of Kayin State [3]. However, in 2021 the number of malaria cases increased significantly again, following the COVID-19 pandemic and the military *coup* which resulted in disruption of health services [4, 5].

The major challenges in controlling and eliminating *P. vivax* are due to its ability to form hypnozoites and cause subsequent relapses, its transmissibility to mosquitoes at an early stage of infection and shorter extrinsic incubation period in the mosquitoes [6–9]. *P. vivax* infections mostly circulate at low parasite densities but can still be transmitted to *Anopheles* mosquitoes [10, 11]. The widely used malaria rapid diagnostic tests (mRDT) that detect parasite antigens have a limited detection threshold (approximately 200 parasites per microliter), therefore missing a substantial number of low-density infections (as well as the liver-stage hypnozoites) that contribute to the continued malaria transmission [12–14]. Previous studies along the Thai-Myanmar border have reported a high prevalence of sub-microscopic *P. vivax* infections up to 25%, highlighting their potential role in sustaining malaria transmission [1, 11, 15].

Radical cure is a critical component of standard case management in the malaria elimination programs. Dual-stage therapy is essential to target both the blood-stage infection and the dormant hypnozoite reservoir, with one component eliminating parasites from the bloodstream (e.g., chloroquine or artemisinin-based combination therapy), while an 8-aminoquinoline drug clears hypnozoites from the liver (e.g., primaquine or tafenoquine) [16]. However, 8-aminoquinolines can induce severe haemolysis in individuals with glucose-6-phosphate dehydrogenase (G6PD) enzyme deficiency. This genetic condition affects up to 30% of people in malaria-endemic areas [17]. The prevalence along the Thai-Myanmar border has been reported to be as high as 18% [18] whereas a study using a different method in Kayin State reported deficiency in 8.9% of the tested individuals [19]. This poses a major barrier to the large-scale implementation of radical cure. In recent years, point-of-care G6PD tests have become available, enabling rapid phenotypic assessment in field settings [20–23]. These tests can be used by the frontline health workers and provide timely results to support the safe administration of primaquine-based radical cure [21, 22].

Mass drug administration (MDA) trials have been successfully deployed as an accelerator tool to eliminate the *P. falciparum* in the presence of generalized access to early diagnosis and treatment and robust surveillance [1, 24]. This principle has been considered for *P. vivax*, where targeting high transmission hotspots requires eliminating the parasite reservoir, including low-density or sub-microscopic infections and treating individuals carrying hypnozoites [25–28]. MDA intervention has limitations related to the population characteristics and drug safety, particularly in people with low G6PD enzymatic activity, pregnant and breastfeeding mothers and younger age groups who cannot receive 8-aminoquinolines [27–30]. Therefore, understanding the burden of G6PD deficiency is essential to design effective intervention strategies for *P. vivax*.

This study examined the prevalence of G6PD deficiency and low-density, sub-RDT *P. vivax* infections in 23 villages in Kayin State, Eastern Myanmar.

## Methods

**Fig. 1:**
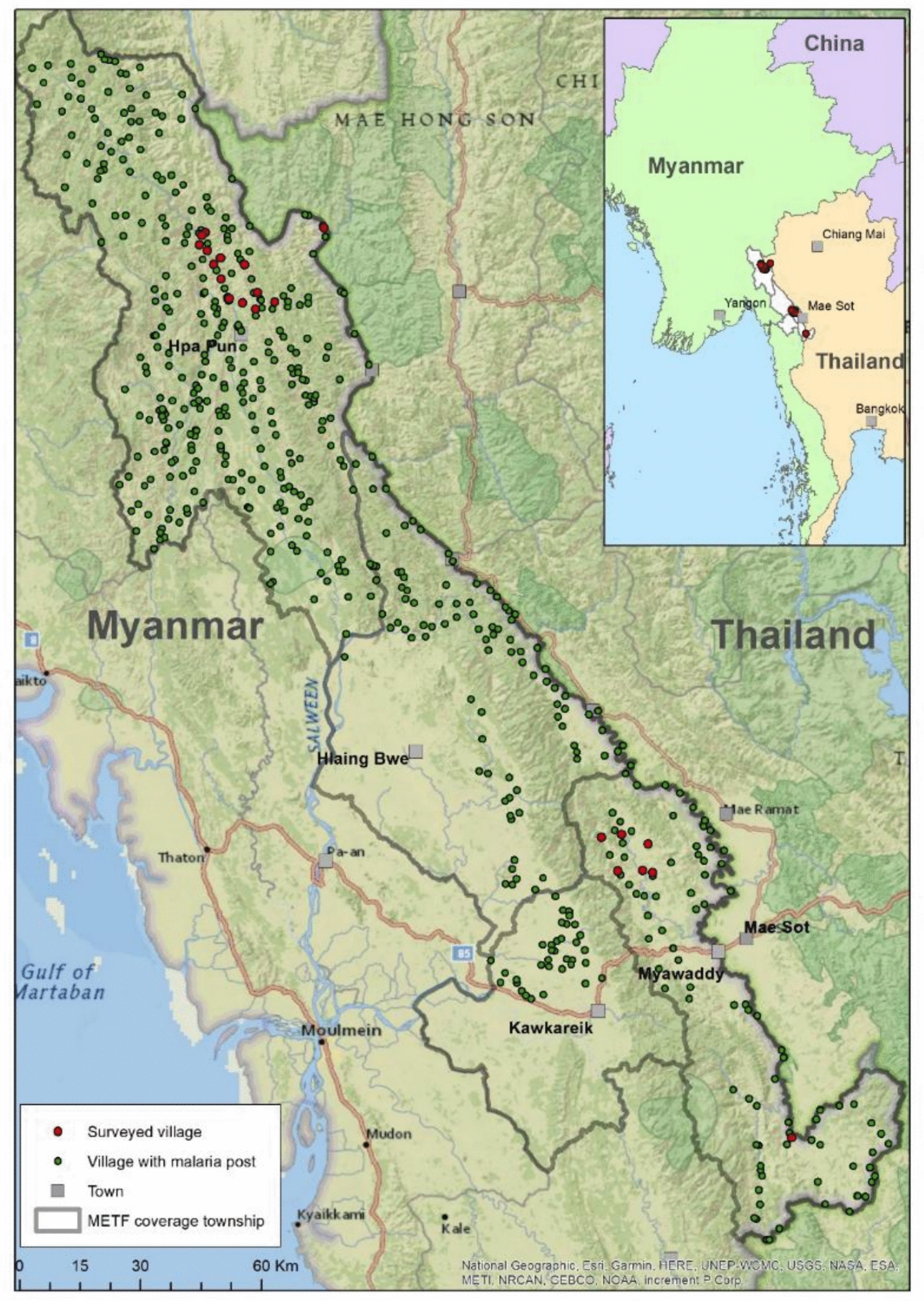
Map of the malaria surveyed villages

Between 2020 (Nov–Dec in 16 villages) and 2021 (May–July in 7 villages), the Malaria Elimination Task Force (METF) program [31] conducted cross-sectional malaria and G6PD screening in 23 villages situated in Kayin State (Fig. 1). Most of these villages (16 out of 23) were in the most remote northern region (Hpapun township) where malaria burden was higher than in the southern region (Myawaddy township) (See supplementary, Figure S1) [3]. Mass malaria and G6PD screenings were conducted using mRDT [Standard Diagnostic, Bioline Pf/Pv Ag that detects the *P. falciparum* histidine-rich protein 2 (Pf HRP2) and *P. vivax*-specific LDH (Pv LDH)] and the “STANDARD^™^ G6PD” test (SD Biosensor, Republic of Korea) was used to measure G6PD enzymatic activity and haemoglobin concentration. Capillary blood (100 µL) was also collected in a microcontainer tube from individuals aged > 5 years who were invited to participate during community engagement sessions and provided informed consent. Enrolment continued until approximately half of the registered village residents had been enrolled. The collected specimens were stored at −80 °C for subsequent molecular and serological analyses. The Q-Plex^™^ Human Malaria Array (5-plex) (Quansys Biosciences, USA) was used to detect Plasmodium antigens. It is a quantitative chemiluminescent enzyme-linked immunosorbent assay (ELISA) allowing concurrent measurement in samples of Pf HRP2, Plasmodium lactate dehydrogenase (Pan LDH), Pv LDH, *Plasmodium falciparum* lactate dehydrogenase (Pf LDH) and C-reactive protein (CRP) using the protocol established by Jang et al. [32]. Highly sensitive, species-specific reverse transcriptase real-time polymerase chain reaction (rRT-PCR) was used for the molecular detection of *P. falciparum* and *P. vivax* Ribonucleic acid (RNA). The rRT-PCR assay for the Genus-specific *Plasmodium* spp. was performed using the primers and probe described in Kamau et al. [33]. Species-specific rRT-PCR assays of *P. falciparum* and *P. vivax* were performed using primer and probe sets described by Wamfler et al. [34], the detailed primers are described in the supplementary text. For species-specific prevalence estimates, *P. falciparum* and *P. vivax* included both mono-infections and mixed (*P. falciparum/P. vivax*) infections. The threshold for G6PD enzymatic activity level was defined as normal (≥ 6.1 U/g Hb in females and ≥ 4.1 U/g Hb in males) and intermediate (4.1–6.0 U/g Hb in females), or deficient (≤ 4.0 U/g Hb in both sexes) according to manufacturer instructions [35]. G6PD results were considered invalid if information on either G6PD enzymatic level or hemoglobin (Hb) values was missing, or if Hb values were lower than < 7.0 g/dL. Children younger than 1 year old were also excluded [35]. Sub-RDT *P. vivax* infections were defined as infections that were negative by mRDT but positive by rRT-PCR.

### Statistical analysis

Descriptive statistics were used to summarize the data. Categorical variables were presented as frequencies and percentages, while continuous variables were summarized using the median and interquartile range (IQR). The positivity rate was defined as the proportion of each diagnostic test performed that were positive across the study population. Village-level prevalence was defined as the proportion of individuals testing positive among the population surveyed in each village. Prevalence estimates with 95% confidence intervals were calculated overall, as well as stratified by sex and geographic area. Differences in proportions across comparison groups were assessed using the chi-square test, with Fisher’s exact test applied when expected cell counts were small. Pf HRP2 and Pv LDH antigen concentrations were right-skewed and were log_₁₀_-transformed. The geometric mean with 95% confidence intervals was calculated for Pv LDH concentration. Pf HRP2 concentrations were summarized using the median and IQR due to the right-skewed distribution of the log₁₀-transformed values. CRP concentrations reported in ng/mL were converted to mg/L. The median values between groups were compared using non-parametric tests. All statistical analyses were performed in R (version 4.4.1). A two-sided p-value < 0.05 was considered statistically significant.

## Results

**Fig. 2:**
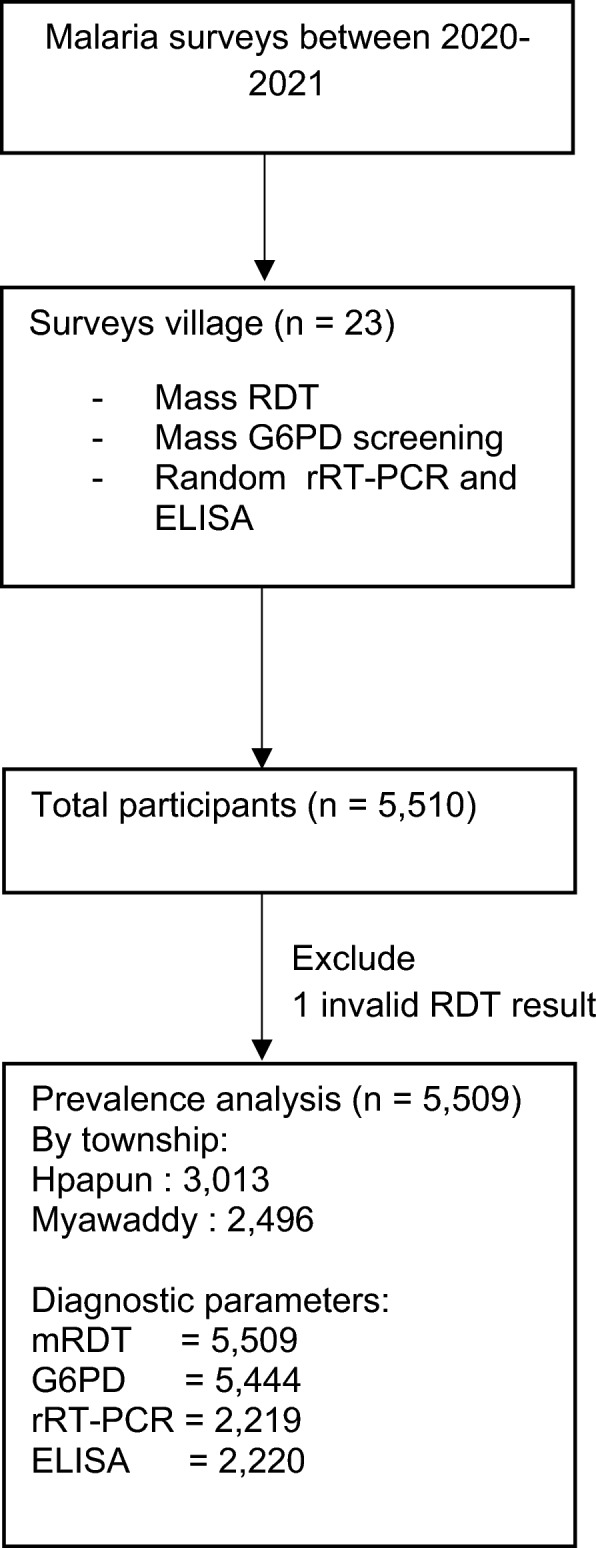
The study analysis flow chart

Prevalence analysis included 5509 participants from 23 villages: 54.6% (3013) of the participants were from Hpapun, and 45.4% (2496) were from Myawaddy township respectively (Fig. 2). The median age was 18 years [interquartile range, IQR 8–35]. Most participants were adults, with females comprising a slightly higher proportion than males (Table 1). Among the participants, all individuals were screened using mRDT and a subset of 2220 individuals (40.2%) underwent both rRT-PCR and ELISA testing; however, rRT-PCR results were available for only 2219 of these individuals, as one sample yielded no result.

**Table 1:** The characteristics of the participants

Characteristics	n = 5509
Age	
Median, years (IQR)	18 (8–35)
Age categories, n (%)	
< 5 years	755 (13.7)
5–15 years	1727 (31.3)
> 15 years	3027 (54.9)
Sex: n (%)	
Female	2914 (52.9)
Male	2595 (47.1)
Residential status^a^	
Villagers	5238 (96.3)
Outsiders	204 (3.7)

^a^67 missing records

### *Plasmodium vivax* prevalence, C-reactive protein and haemoblogin

The malaria positivity rate by mRDT was very low (< 1%), with 26 *P. vivax* and 1 *P. falciparum* infections detected among 5509 individuals. The overall rRT-PCR positive rate was highest for *P. vivax*, 14.3% (317/2219) followed by *P. falciparum*, 8.3% (185/2219) and unidentified *Plasmodium *species infections, 0.8% (18/2219) (Table 2). The positive rate by ELISA was approximately 19-fold higher than mRDT (9.5% vs 0.5%), yet lower than detected by rRT-PCR (14.3%) (Table 2). ELISA assay detected about half (160/317) of *P. vivax* rRT-PCR confirmed infections (Table S1).

**Table 2:** Malaria prevalence by RDT, rRT-PCR and ELISA by age group and sex

Type of diagnosis	Number tested	Positivity, %	Age distribution of positives, %			Sex distribution of positives, %	
			< 5 years	5 to 15 years	> 15 years	Male	Female
RDT	5509						
*P. falciparum*	1	0.1	–	–	100.0	–	100.0
*P. vivax*	26	0.5	19.2	57.5	23.1	65.4	34.4
rRT-PCR	2219						
*P. falciparum*	185	8.3	–	20.0	80.0	53.0	47.0
*P. vivax*	317	14.3	–	17.4	82.6	56.5	43.5
^a^*Plasmodium* spp.	18	0.8	–	11.1	88.9	72.2	27.8
ELISA	2220						
P.f HRP2	22	1.0	–	22.7	77.2	40.9	59.1
P.f LDH	29	1.3	–	31.0	69.0	58.6	41.4
P.v LDH	212	9.5	–	19.3	80.7	58.9	41.1
Pan LDH	271	12.2		16.6	83.4	59.0	41.0

^a^Species cannot be identified

**Fig. 3:**
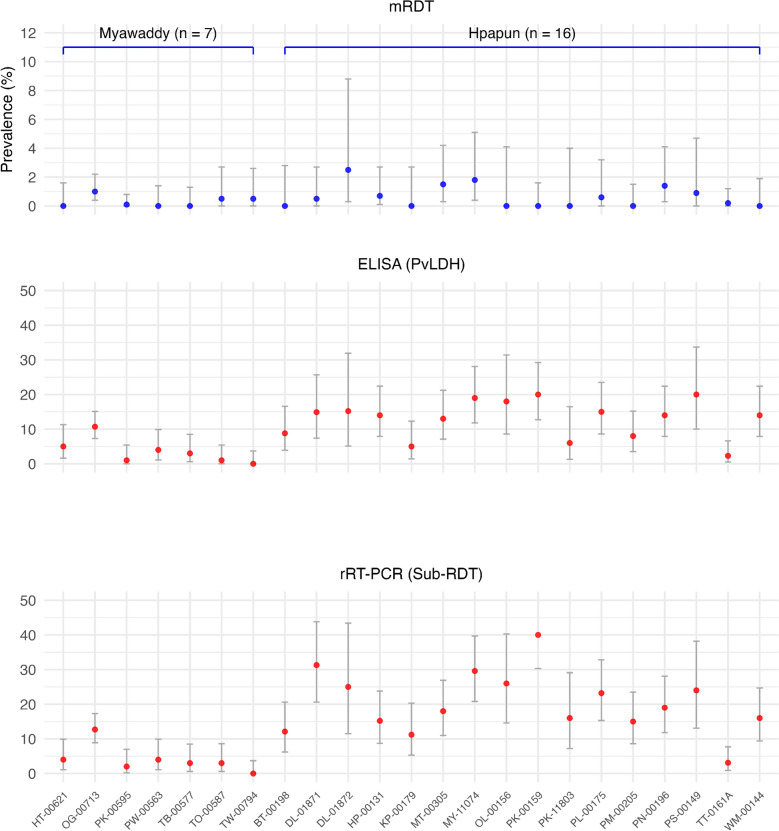
*P. vivax* prevalence per village measured by mRDT, Pv LDH ELISA, and sub-RDT (rRT-PCR), arranged by townships; Myawaddy (south) and Hpapun (north)

Sub-RDT *P. vivax* infections were detected in 14.1% (311 of 2211) individuals after excluding eight mRDT positive infections. At the village level, the median prevalence of sub-RDT *P. vivax* infections was 15.2% [IQR 4.0–23.6, range 0–40.4] (Fig. 3). The prevalence was six-fold higher in villages located in Hpapun township, compared to those in Myawaddy township (Figure S2). In contrast, *P. vivax* village level prevalence identified by mRDT was very low (median 0%, [IQR 0–0.3, range 0–2.0]) (Fig. 3). The median village level prevalence of Pv LDH was 10.7% [IQR 4.5–14.9, range 0–20%] (Fig. 3).

The overall median CRP value was 0.2 [IQR 0.1–0.5; min–max 0.1–28.8]. In Myawaddy township, median CRP levels were higher in sub-RDT *P. vivax* positives (0.5 mg/L [IQR 0.1–1.7]) than in negative individuals (0.2 mg/L [IQR 0.1–0.4]) (P < 0.001). However, no significant difference in CRP values was observed between *P. vivax* sub-RDT positive and negative groups in Hpapun township (P = 0.5) (Table S2).

Mean haemoglobin concentrations were compared across *P. vivax* rRT-PCR positive and negative groups within townships and sex strata (Table S3). No clinically meaningful differences were observed.

### *Plasmodium falciparum* prevalence

The *P. falciparum* positivity rate by rRT-PCR was 8.3% (185/2219). In comparison, the ELISA assay targeting Pf HRP2 detected 1.0% of the tested participants (22/2220), while mRDT detected only one case (Table 2). The overall prevalence of sub-RDT *P. falciparum* infections was 8.3% (184/2218). At the village level, the median prevalence was 6.0% (IQR 2.1–16.0; range 0–27.0) (Figure S3).

### Age and sex

The detection of *P. vivax* by mRDT was highest in the young age group (5–15 years) at 57.5% (15/26), and was higher among males at 65.4% (17/26) (Table 2). In contrast, rRT-PCR detection showed that the majority of *P. vivax* infections occurred in the older age group (> 15 years), accounting for 82.6% (262/317) of cases (Table 2). Among sub-RDT *P. vivax* infections, the proportion was also highest in those aged > 15 years (83.6%, 260/311). A similar age and sex-specific distribution was observed for *P. vivax* prevalence measured by Pv LDH ELISA (Table 2). Among individuals with rRT-PCR confirmed *P. vivax* infections, the geometric mean of Pv LDH level was higher in the younger age group (156.2 pg/mL, [95% CI 75.9–321.0]) than in adults (72.6 pg/mL, [95% CI 54.4–96.8]), though the difference was not statistically significant (p = 0.051) (Figure S4).

Among *P. falciparum* infections, the positivity rate was higher in the older age group (> 15 years) as detected by rRT-PCR and Pf HRP2 ELISA (Table 2). However, Pf HRP2 antigen concentrations did not differ significantly between those age > 15 years (median = 0.7 pg/mL [IQR 0.7–0.8]) and those aged < 15 years (median 0.7 pg/mL [IQR 0.7–0.9] (p = 0.44) among rRT-PCR positives.

### G6PD deficiency prevalence

**Fig. 4:**
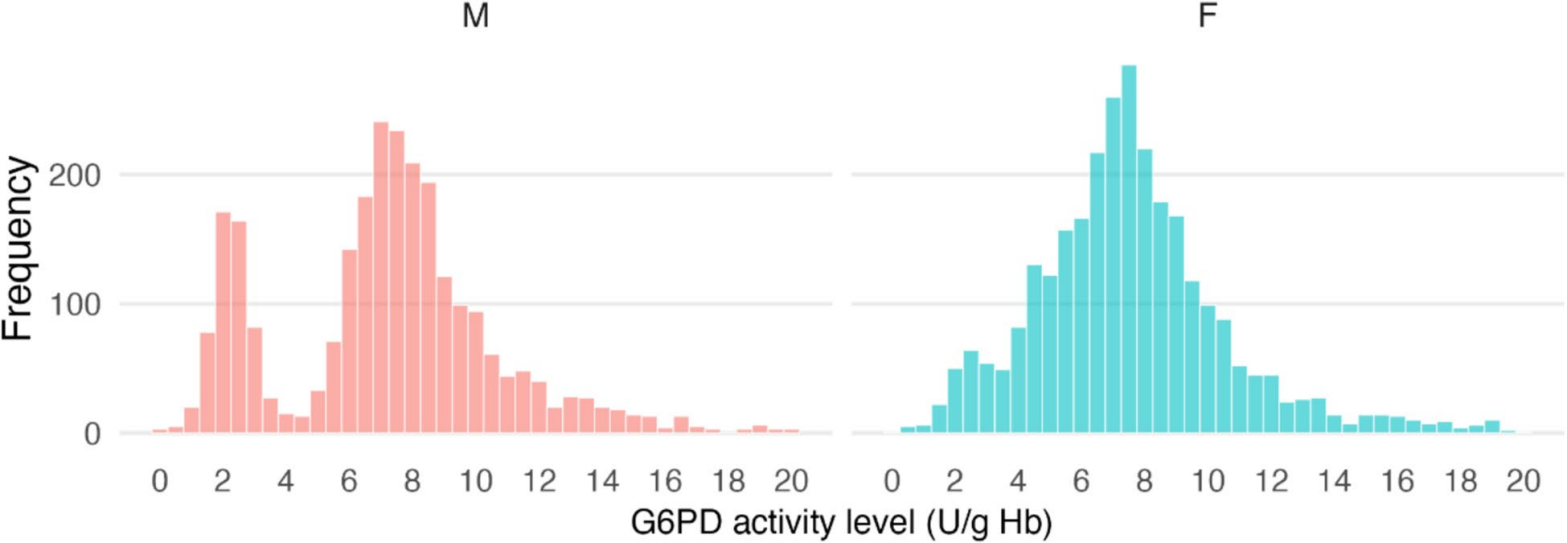
The distribution of G6PD enzymatic activity (U/g Hb), (left) male and (right) female

In the participants to the surveys, quantitative G6PD test results were available for 5444 (98.8%) of the 5509 participants. The median G6PD enzymatic activity was similar among males and females: 7.3 U/g Hb [IQR 5.6–8.9] and 7.3 U/g Hb [5.7–9.0] (Fig. 4). The overall prevalence of G6PD deficiency was 15.8% [95% CI 14.8–16.8]. The proportion of deficiency was higher in males (21.7%) compared to females (10.6%) (P < 0.001) while the intermediate activity levels among females were 18.6%. The prevalence of G6PD deficiency varied across villages with a median prevalence in Myawaddy of 14.4% [IQR 12.3–14.8] and 17.3% [IQR 14.0–23.1] in Hpapun township (P = 0.05) respectively (Figure S5).

### Sub-RDT *P. vivax* infection and G6PD deficiency

Of 2219 individuals tested by rRT-PCR, 63 were excluded (9 *P. vivax* RDT positives, 22 rRT-PCR *P. falciparum* mono-infection, 18 indetermined plasmodium species, and 14 invalid G6PD results). The final group included 2156 participants with valid rRT-PCR and G6PD results for comparison of sub-RDT *P. vivax* positivity across G6PD categories. The proportion of sub-RDT *P. vivax* positive infections differed across G6PD categories: 19.2% (69/359) in G6PD-deficient, 13.9% (32/230) in intermediate, and 13.4% (209/1567) in G6PD-normal participants (P = 0.02). However, when stratified by sex, no statistically significant association was observed between sub-RDT *P. vivax* infection and G6PD status among either males or females. This lack of association was consistent across the different townships (Table 3).

**Table 3:** Sub-RDT *P. vivax* infection by G6PD status, stratified by sex and township

Sex	G6PD status	Sub-RDT *P. vivax* negative, n (%)	Sub-RDT *P. vivax* positive, n (%)	Chi-square p-value
A. Hpapun				
Male	Normal	323 (81.9)	105 (18.1)	0.51
	Deficient	104 (79.6)	40 (20.4)	–
Female	Normal	421 (86.6)	65 (13.4)	0.08
	Intermediate	120 (81.1)	28 (18.9)	–
	Deficient	86 (79.6)	22 (20.4)	–
B. Myawaddy				
Sex	G6PD status	Sub-RDT *P. vivax* negative, n (%)	Sub-RDT *P. vivax* positive, n (%)	p-value
Male	Normal	265 (90.8)	27 (9.2)	0.46
	Deficient	65 (94.2)	4 (5.8)	–
Female	Normal	349 (96.7)	12 (3.3)	0.35
	Intermediate	78 (95.1)	4 (4.9)	–
	Deficient	35 (92.1)	3 (7.9)	–

## Discussion

In Myanmar, malaria cases declined markedly between 2014 and 2020 following the intensified malaria elimination efforts, including those in Kayin State [1, 2]. As transmission declined, *P. vivax* became predominant [1]. Elimination of *P. vivax* remains difficult due to radical cure contraindications and the presence of hidden reservoirs.

In these malaria surveys, the mRDT detection rate for *P. vivax* was very low whereas the prevalence of sub-RDT infections (detected by Pv LDH ELISA and rRT-PCR) was substantially higher. This discrepancy is expected, as mRDT typically have detection limits of approximately 100–200 parasites per µL and were not designed to detect low-density infections. In comparison, the ELISA assay identified approximately half of the rRT-PCR confirmed cases for *P. vivax*, which was greater than the detection rate observed with mRDT. This enhanced sensitivity of ELISA can be attributed to its lower limit of detection for Pv LDH antigen concentration than commercially available mRDTs [32, 36]. The rRT-PCR method used in this study had higher sensitivity and a lower limit of detection than other molecular methods such as ultrasensitive PCR (uPCR) circa: 0.022 parasitise per µL [37]. This increased analytical sensitivity allows detection of very low parasite densities, which likely explains why antigen-based ELISA assays fail to identify low density malaria infections where antigen concentrations fall below the detectable thresholds. Additionally, variability in circulating Pv LDH blood concentration may affect results, as levels fluctuate and do not consistently correlate with parasite density [36]. The 5-Plex ELISA assay was compared to rRT-PCR because it offers a high-throughput, multiplex platform suitable for large-scale malaria programs. Although rRT-PCR delivers better analytical sensitivity and can detect low density sub-RDT infections, its complexity, cost, and laboratory needs make it impractical for routine use. However, in this study, rRT-PCR showed much higher sensitivity than ELISA and identified infections that antigen-based malaria screening methods would miss. The sub-RDT *P. vivax* carriage rate was higher in Hpapun township, where the number of clinical malaria cases was reported to be higher than in Myawaddy [3]. This difference probably reflects the higher transmission of malaria in the northern area, translating in a higher clinical caseload. The season of sampling may have had a small effect since the northern villages were sampled during the rainy season while the southern ones were surveyed in the dry season. Travel history was not explicitly recorded; however, over 96% of participants were primary residents of their surveyed villages, reflecting a largely settled population. Additionally, the low-density infections detected here differ fundamentally from the acute, high-density parasitaemia typically linked to recent travel [3]. Imported infections are therefore unlikely to contribute substantially to this reservoir.

Current diagnostic tools cannot directly detect *P. vivax* hypnozoites in the liver. Antibody-based serological assays are under development as proxy diagnostic tool to detect hypnozoites carriers [38], with feasibility of field application shown in Cambodia [39]. While these methods may identify likely hypnozoite carriers, the present study focused on detecting low-density infections using rRT-PCR and antigen-based ELISA, thus, antibody detection was limited by diagnostic method applied. *P. vivax* serological testing and treatment approach has yet to be evaluated in high-transmission settings such as Kayin State, Myanmar, where *P. vivax* is prevalent and malaria has recently resurged [5]. In these context, repeated parasite exposure can induce prolonged antibody responses, potentially reducing test specificity [40]; therefore, the effectiveness of this approach requires further assessment.

A difference in CRP levels, an acute-phase inflammatory marker, was observed between individuals with sub-RDT *P. vivax* infection and non-infected participants. However, CRP concentrations in both groups remained below established clinically relevant thresholds and were also lower than those reported in a previous study of low density *P. vivax* carriage [41]. The use of a more sensitive rRT-PCR with a lower limit of detection in present study likely captured lower-density *P. vivax* infections with minimal inflammatory responses and thus lower CRP levels. Prior evaluations using 5-plex ELISA assays have shown that CRP elevations are more pronounced in febrile malaria and have limited discriminatory value in low-density infections [32, 42].

Distinct age-specific patterns of *P. vivax* infection were observed across diagnostic methods. The mRDT-positive infections were more commonly detected among children aged 5–15 years, reflecting higher parasite densities. In contrast, rRT-PCR and ELISA identified a greater number of infections among adults, reflecting low-density persistent infections likely maintained by partial immunity from repeated exposure which remain undetectable by mRDT [43, 44]. These sub-RDT infections in adults were asymptomatic, unlikely to prompt care seeking and therefore missed by passive surveillance despite constituting a substantial reservoir of infection [45]. In addition, working age men and women are more mobile and increasing the potential for parasite dissemination across villages and transmission clusters [46]. As a result, surveillance systems relying on mRDT-based case detection systematically miss this adult reservoir and fail to trigger appropriate response measures targeting these infections.

Previous surveys in Kayin State (2014–2018) reported a similar prevalence of low-density *P. vivax* infections [1]. These surveys conducted between 2020 and 2021 confirm the continued presence of low density *P. vivax* reservoir despite years of early detection and treatment intervention through community malaria posts. Radical cure of *P. vivax* had not yet been widely implemented during this period [1, 31] and prior MDA campaigns targeting *P. falciparum* in Kayin State had minimal effect on *P. vivax* [1]. In addition, dominant *Anopheles* vectors in Kayin State exhibit early and outdoor biting behaviour [10], limiting the effectiveness of long-lasting insecticide-treated nets (LLINs) and indoor residual spraying (IRS). Together, these factors indicate that passive case detection and conventional vector control alone are insufficient to rapidly eliminate *P. vivax* where a substantial proportion of infections remain low-density and untreated. Targeting sub-microscopic and sub-RDT reservoirs is therefore essential to accelerate elimination.

The prevalence of G6PD deficiency in Myanmar varies widely across geographical regions and ethnic groups. Previous studies reported rates of 10.8% in central Myanmar [47] and 5.6 to 10.0% in the western region [48, 49]. The deficiency was reported to be more common among Kachin and Karen people compared to other ethnic groups [18, 50, 51]. In this study, the overall prevalence of G6PD deficiency was 15.8%, reinforcing existing evidence of a substantial burden among populations living in Kayin State.

The high prevalence of G6PD deficiency observed here suggests that a considerable proportion of the population would be ineligible for shorter and higher dose primaquine or tafenoquine regimens, potentially limiting the applicability of the regimens that are designed to improve adherence to radical cure. When considering MDA or community-wide radical cure campaigns, additional exclusions related to pregnancy, breastfeeding, and age restrictions would further limit population coverage and overall effectiveness [52]. Strategic selection of radical cure regimens should be considered for populations with varying G6PD status to maximize effectiveness. In addition, *P. vivax* elimination strategies must address populations who are not eligible for standard 8-aminoquinoline therapy. Shorter ascending-dose primaquine regimens represent a potential alternative for G6PD-deficient individuals but require further validation in clinical settings prior to community-level implementation. [53].

Evidence from several regions suggests that the G6PD deficiency can confer protection against clinical severe malaria [54–56]. Earlier studies reported reduced symptomatic *P. vivax* incidence among G6PD-deficient individuals with the Mediterranean variant [56] and along the China-Myanmar border, the Mahidol variant was associated with protection against acute *P. vivax* infections [51]. However, these studies had very different designs and focussed on the protection against clinical *P. vivax* malaria whereas the study presented here looked at asymptomatic low-density infections. In the present study the prevalence of sub-RDT *P. vivax* infections was higher in the G6PD deficient participants, and no association was observed between sub-RDT *P. vivax* infections and G6PD status.

Although this study primarily focuses on *P. vivax*, it is also important to consider the epidemiological trends of *P. falciparum* in the same setting. The clinical incidence of *P. falciparum* malaria declined substantially between 2014 and 2020, however, low-density infections remained detectable particular in the northern area. This likely reflects the higher sensitivity of the rRT-PCR method used in this survey, together with the resurgence of malaria following the COVID-19 pandemic and the ensuing disruption of services [3, 5].

## Limitation

G6PD variant genotyping was not performed in this study, therefore, associations between specific genotypes and low-density or sub-RDT infections could not be assessed. In addition, children under five years of age were excluded from additional blood specimen collection, as predefined in the study design. Malaria blood smear was not collected, and the parasite count could not be measured by microscopy, which limited the assessment of its relationships with rRT-PCR results and antigen concentrations by ELISA assay.

## Conclusion

The presence of sub-RDT *P. vivax* infections and G6PD deficiency add significant complexity to control and eliminate *P. vivax* malaria in Kayin state, Myanmar. This dual challenge underscores the need for high-sensitive diagnostic tools to detect low-density infections and robust G6PD point-of-care testing to ensure safe and effective radical cure implementation.

## Supplementary Information

Supplementary Material 1.

## Data Availability

The data utilized for this study are available upon request to the Mahidol-Oxford Tropical Medicine Research Unit data access committee (datasharing@tropmedres.ac).

## Abbreviations

METF: Malaria Elimination Task Force
G6PD: Glucose 6 Phosphate Dehydrogenase
mRDT: Malaria Rapid Diagnostic Test
rRT-PCR: Reverse transcriptase real-time polymerase chain reaction
RNA: Ribonucleic acid
HRP2: Histidine Rich Protein 2
LDH: Lactate Dehydrogenase
*P. falciparum*: *Plasmodium falciparum*
*P. ** vivax*: *Plasmodium vivax*
