# Supplementary material for: Sub-RDT Plasmodium vivax infections and G6PD deficiency in Kayin State, Myanmar
Source: Malar J. 2026 Jun 1;25:240. doi: 10.1186/s12936-026-05952-7 (PMC13281254; doi:10.1186/s12936-026-05952-7)
Supplement: Supplementary file 1 — Supplementary Material 1. [file 12936_2026_5952_MOESM1_ESM.docx]

**Supplementary figures and tables**

**
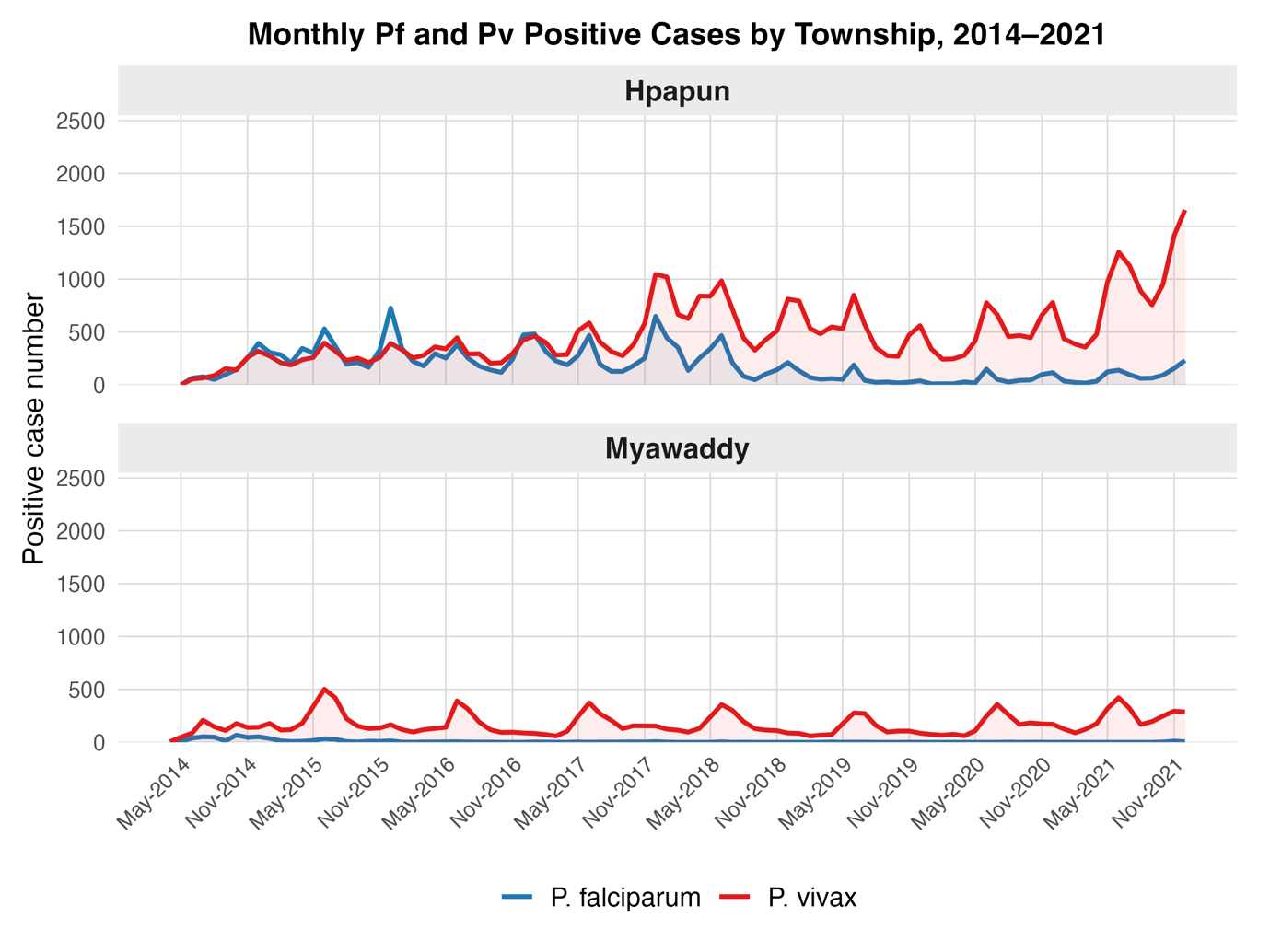
**

Figure S1 Monthly malaria cases from 2014 to 2021 in Hpapun and Myawaddy, Kayin State, Myanmar.


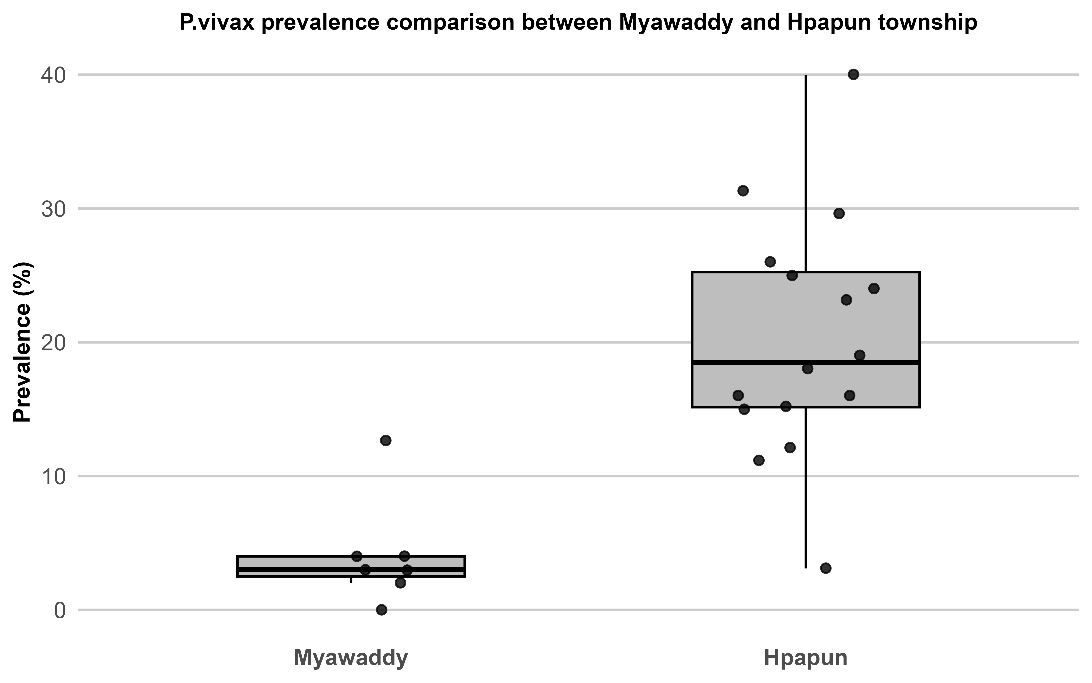


Figure S2 Sub-RDT *Plasmodium vivax* prevalence in Myawaddy (left) and Hpapun (right) townships. Boxplot showing the median and interquartile range.


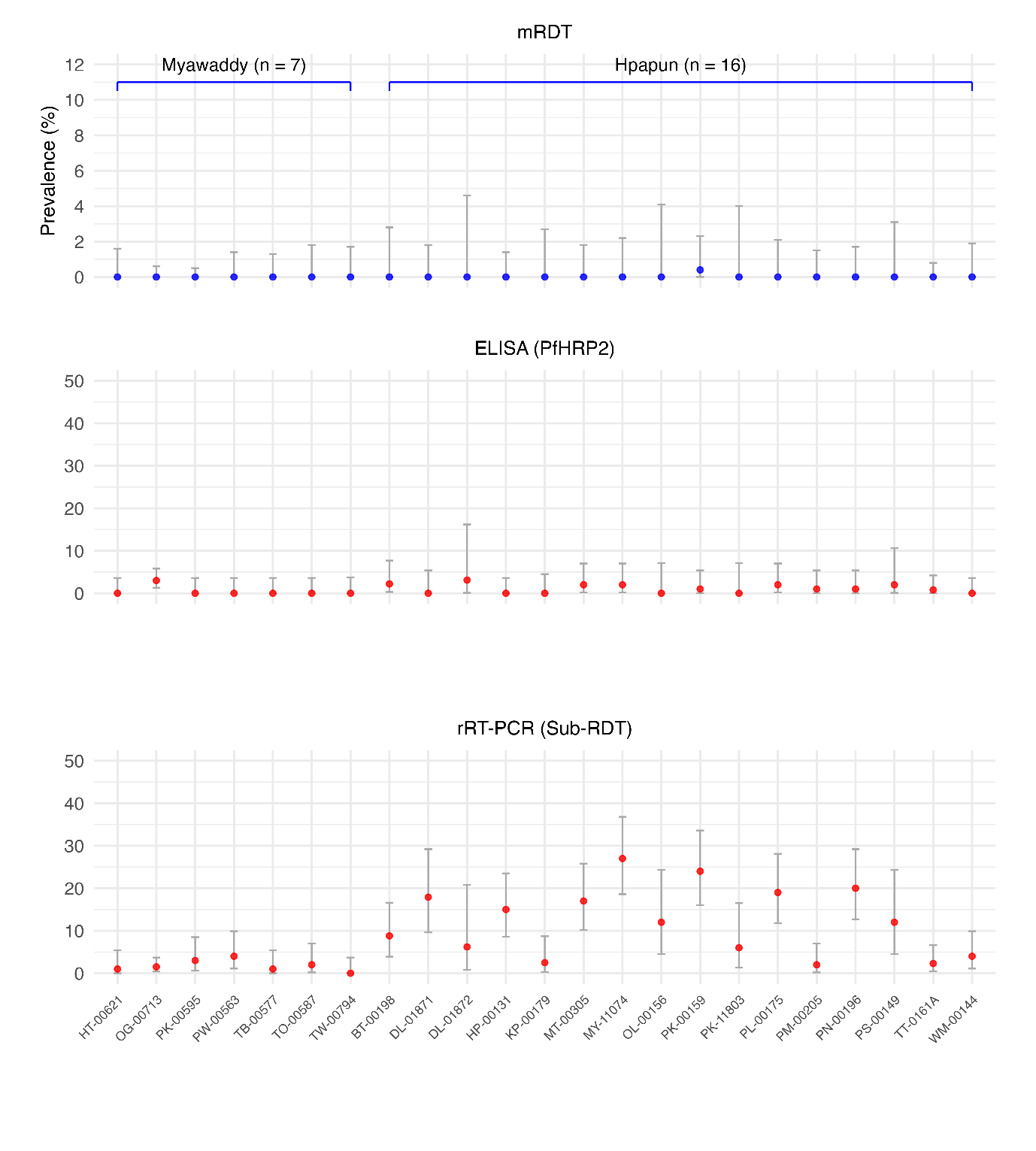


Figure S3. *P. falciparum* prevalence by village measured by mRDT, Pf HRP2 ELISA, and sub-RDT (rRT-PCR), arranged by townships; Myawaddy (south) and Hpapun (north).


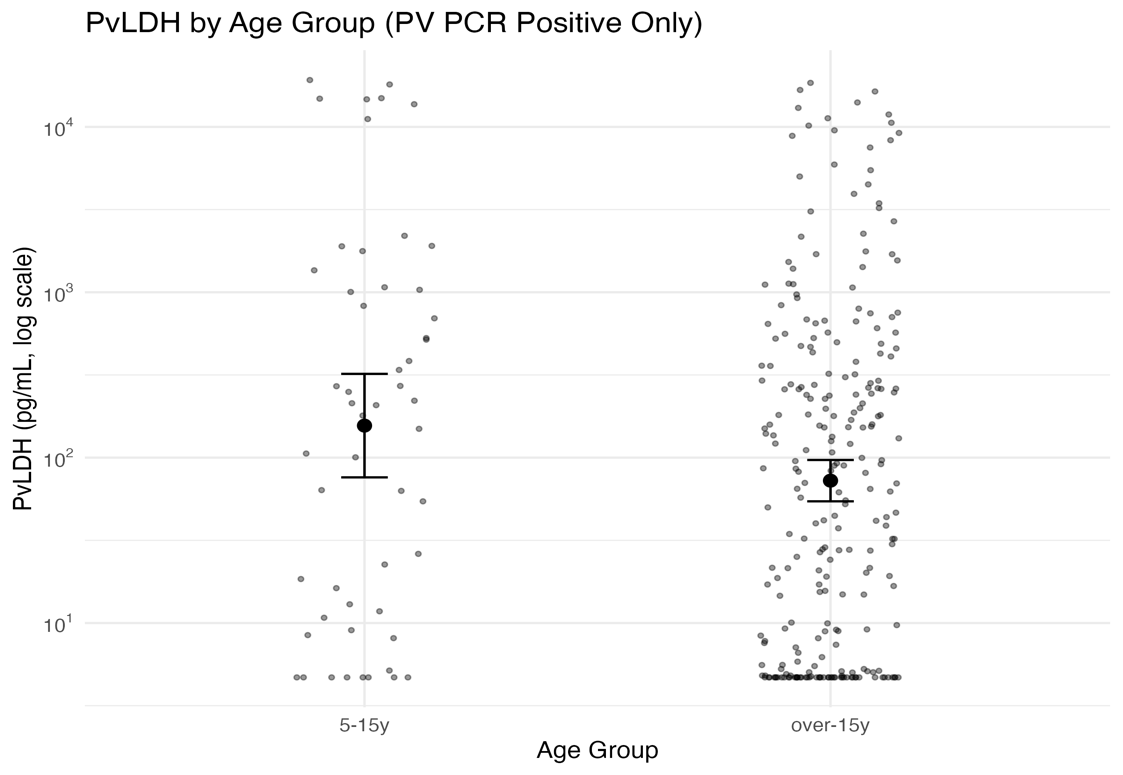


Figure S4. Geometric mean PvLDH levels with 95% confidence intervals by age group among rRT-PCR–confirmed *Plasmodium vivax* infections


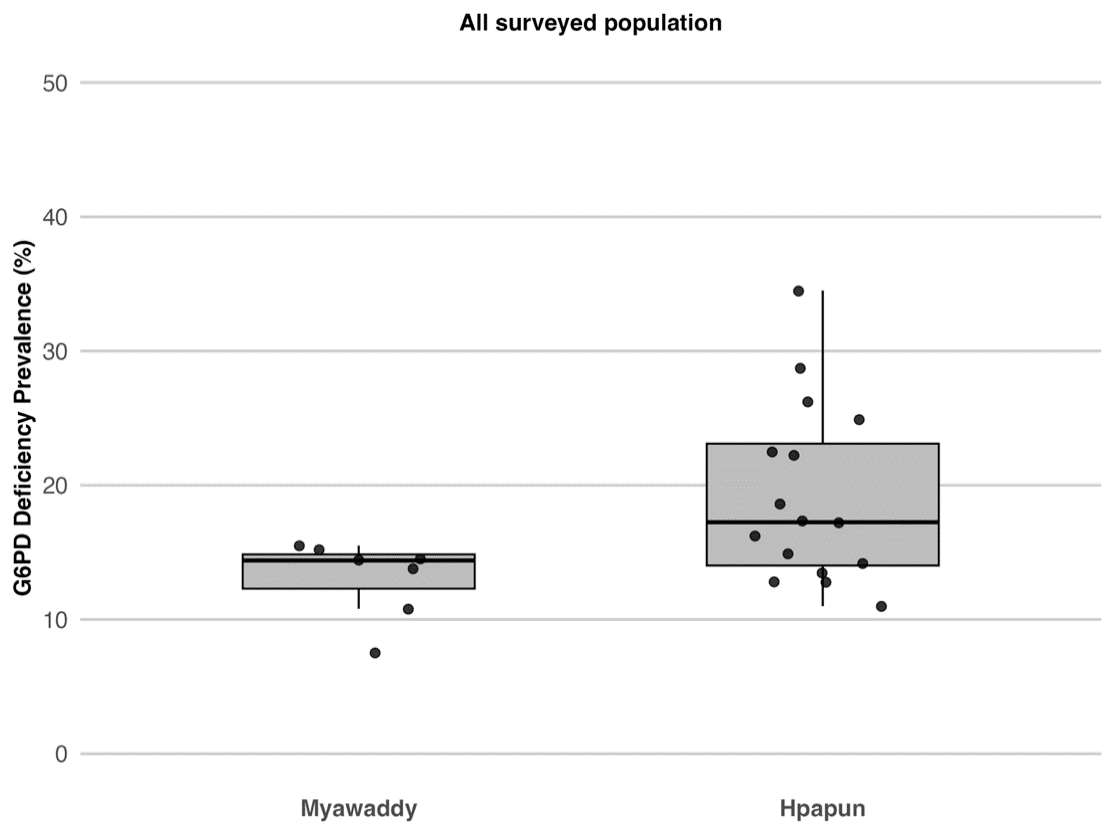


Figure S5. G6PD deficiency prevalence in Myawaddy and Hpapun townships. Boxplot showing the median and interquartile range.

Table S1. The proportion of infections detected by ELISA among the rRT-PCR positive *P. falciparum* and *P. vivax* infections.

|  | rRT-PCR (*P. falciparum*) | | |
| --- | --- | --- | --- |
| 5-plex ELISA  *P. falciparum* | Positive  n = 185 | Negative  n = 2,034 | Total |
| Pf HRP2 positive, n (%) | 7 (3.8%) | 15 (0.7%) | 22 |
| Pf LDH positive | 17 (9.2%) | 12 (0.6%) | 29 |
| rRT-PCR (*P. vivax*) | | | |
| 5-plex ELISA  *P. vivax* | Positive  n = 317 | Negative  n = 1,902 | Total |
| Pv LDH positive | 160 (50.5%) | 52 (2.7%) | 212 |

Table S2 Median CRP levels in participants with different *P. vivax* infection status across Hpapun and Myawaddy townships.

| Township | N* | **Sub-RDT *P. vivax*** | **CRP level (mg/L)  median (IQR), [min-max]** |
| --- | --- | --- | --- |
| Hpapun | 1,047 | NEG | 0.2 (0.1 - 0.5), [0 – 28.7] |
| Hpapun | 259 | POS | 0.2 (0.1 – 0.6), [0 – 23.8] |
| Myawaddy | 794 | NEG | 0.2 (0.1 – 0.4), [0 – 28.1] |
| Myawaddy | 48 | POS | 0.5 (0.1 – 1.7), [0 – 27.7] |

*CRP values not available in 23 out 2110 sub-RDT *P. vivax* result

Table S3 Mean haemoglobin concentrations by township, sex, and *P. vivax* sub-RDT status

| Township | Sex | Sub-RDT *P. vivax* negative  Hb result (Hb in g/dl mean ± SD) | Sub-RDT *P. vivax*  positive  Hb result (Hb in g/dl mean ± SD) |
| --- | --- | --- | --- |
| Hpapun | F | 11.8 ± 1.6 | 11.4 ± 1.4 |
| Hpapun | M | 13.5 ± 1.8 | 13.8 ± 1.6 |
| Myawaddy | F | 12.6 ± 1.4 | 12.1 ± 1.5 |
| Myawaddy | M | 14.1 ± 1.7 | 14.1 ± 1.8 |

**Supplementary text**

This table lists the primers and probes used for the molecular detection of malaria parasites, targeting three species: Plasmodium spp. (genus-level detection), *P. falciparum*, and *P. vivax.* Each entry includes the primer/probe name, the nucleotide sequence in 5'→3' orientation, and the corresponding published reference.

| **Species** | **Primer/probe name** | **Sequence (5' →3')** | **Reference** |
| --- | --- | --- | --- |
| *Plasmodium* spp. | PreFor | GCTTTCTTGATTTCTTGGATG | [33] |
|  | PreRev | AGCAGGTTAAGATCTCGTTCG |  |
|  | PreProBHQProbe | FAM-ATGGCCGTTTTAGTTCGTG-BHQ1 |  |
| *P. falciparum* | PF_A18S_fw | TCCGATAACGAACGAGATCTTAAC | [34] |
|  | PF_A18S_rv | ATGTATAGTTACCTATGTTCAATTTCA |  |
|  | PF_A18S_probe | FAM-TAGCGGCGAGTACACTATA-MGB |  |
| *P. vivax* | PV_18S_fw | GCTTTGTAATTGGAATGATGGGAAT | [34] |
|  | PV_18S_rv | ATGCGCACAAAGTCGATACGAAG |  |
|  | PV_18S_probe | *HEX-AGCAACGCTTCTAGCTTA -MGB |  |
